# Cloning CTL-Specific Genes (And Now for Something Completely Differential)

**DOI:** 10.3389/fimmu.2014.00509

**Published:** 2014-10-14

**Authors:** R. Chris Bleackley

**Affiliations:** ^1^Department of Biochemistry, University of Alberta, Edmonton, AB, Canada

**Keywords:** granzymes, proteases, immunology, gene expression, cytotoxic T lymphocyte, natural killer cells

The work described here began as a project to use differential hybridization to identify genes that were induced after T cell activation. In reality, it became something completely different when the approach identified genes that encoded proteins involved in the killing mechanism used by T lymphocytes (1, 2).

The late 1970s and early 1980s were exciting times of discovery in immunology. Lots of fascinating biological observations had already been made but there was little in the way of molecular understanding. During this period, the newly emerging tools of recombinant DNA technology and molecular biology were becoming more readily accessible and being applied to study immunological questions. This led to many exciting discoveries such as antibody and T cell receptor gene rearrangements (3–5).

I had some experience looking at antibody genes and the biochemical and genetic characterization of interleukin 2. Using these new approaches, I decided to investigate cytotoxic T cell (CTL) activation at the level of gene activation. The guiding hypothesis was that the phenotype of a cell was governed by the proteins expressed. We posited that each cell would express a set of function related proteins and their corresponding mRNAs. Consequently, we set about to identify mRNAs that were expressed specifically in activated CTL. At worst, we believed that this approach would uncover a set of interesting genes to study CTL activation at the transcriptional level. The real hope was that we would identify proteins involved in the killing mechanism. Interestingly, when this was reviewed for funding it was described as a fishing expedition. These days that would be the kiss of death for a proposal. However, these were more reasonable times and it was funded.

At the time, very little was known about how CTL induced death in target cells that they recognized. The effector cells were known to contain cytoplasmic granules that polarized toward the target upon conjugate formation. It was believed that they contained cytolytic proteins that induced membrane damage. One of the key molecules responsible for the ring-like lesion observed on target cells was ultimately shown to be cytolysin/perforin (6, 7).

We took advantage of the discovery of methods for the long term culture of CTL (8) and had generated a CTL line MTL2.8.2 (9). Using mRNA from this killer cell, a library of 4000 cDNA clones were generated. Individual colonies were picked and isolated in microtiter plates. Copies of these were replicated in triplicate onto nitrocellulose and grown. After lysis, the blots were hybridized with 32-P cDNA probe generated from MTL poly A^+^ RNA. Positive colonies were identified and then the three copies of the library on the membrane were stripped and re-probed with radioactive cDNA prepared from a helper T cell line CH1. After autoradiography, the blots were again stripped and hybridized with probe from unactivated thymocytes.

The various autoradiograms were compared. Only colonies, which were strongly positive in two copies with the MTL probe and negative with CH1 and thymocyte cDNA, were selected. That gave us 121, which were rescreened yielding 36, which were clearly CTL specific. Two were chosen for further analysis; B10 was the most abundant and cross hybridized with eight other inserts and C11 because it was related to B10 but different. Tissue specificity cytodots clearly established that these two were CTL specific. The one exception was a signal in a suppressor T cell line derived from fetal thymus (10). This was not pursued further as the suppressor field was murky at best at this time. However, now it seems likely that this was a regulatory T cell.

The key observation was the correlation of mRNA expression of both B10 and C11 with cytotoxicity in both allogeneic and mitogen stimulated spleen cells. The level of killing measured in a chromium release assay peaked roughly 24 h after the maximum levels of both B10 and C11. In addition, as cytotoxicity declined, so did expression of the mRNAs (1). This was exactly the behavior expected for function related transcripts when it became very exciting.

A full length clone for C11 was isolated and sequenced. An open reading frame was identified, which predicted that the protein encoded was a serine proteinase most similar to the rat mast cell proteinases. Most important, the predicted protein contained the catalytic triad responsible for the enzymatic activity of this family of enzymes. A full length clone corresponding to B10 could not be found but sequence homology and sequence alignment predicted that it also encoded a proteinase.

At this time, I met Irv Weissman at a conference and discovered that his group had also cloned a serine proteinase gene from activated CTL. Sequences were compared and much to our delight that they were related but different. It was decided that the two manuscripts would be submitted together to Science. Rather surprisingly, Irv’s paper was accepted but ours was rejected. Only through Irv’s direct intervention with the editor was this decision reversed and the two papers appeared side-by-side (2, 11).

They named their enzyme Hanukkah Factor while ours were christened cytotoxic cell proteinase 1 and 2 (CCP). Following the outstanding biochemical purification of a family of serine proteinases from CTL granule by Jurg Tschopp’s group, they became more commonly called granzymes (12). Granzyme B (CCP1) was also named CTLA1 by Pierre Goldstein’s group (13). The majority of these genes are clustered on mouse chromosome 14 close to the alpha-chain of the TCR (14).

The discovery of these enzymes created quite a stir of excitement in the field. Hudig and Redelman had suggested, for many years, a key role for proteinases in the killing mechanism. Pasternack et al. had recently demonstrated the importance of a trypsin-like enzyme that was associated with perforin containing granules and secreted upon target cell interaction (15). In addition, the groups of Podack and Henkart had purified the proteins responsible for the ring-like lesions seen in membranes treated with cytolytic granules. They named the molecules perforin (6) and cytolysin (7), but later it became apparent that they were the same protein. The general consensus was that the killing mechanism involved a proteinase cascade analogous to that seen in the complement system. The ultimate target of this cascade model was perforin, which upon activation would create the membrane damage observed.

We now know that perforin mediated target cell lysis is not the whole story. Rather cells under attack by CTL die via a mechanism involving DNA fragmentation. Perforin alone cannot mediate this form of death. It is now known that perforin facilitates the uptake and release of granzymes into the cytoplasm of the targets. The granzymes then cleave specific substrates that bring about the ultimate demise of the cell. Most notable is the cleavage of caspases by granzyme B that initiates apoptosis and brings about DNA fragmentation, but it took another 10 years to discover this (16, 17).

## Conflict of Interest Statement

The author declares that the research was conducted in the absence of any commercial or financial relationships that could be construed as a potential conflict of interest.
